# Unlocking Patients with Mental Disorders Who Were in Restraints at Home: A National Follow-Up Study of China’s New Public Mental Health Initiatives

**DOI:** 10.1371/journal.pone.0121425

**Published:** 2015-04-07

**Authors:** Lili Guan, Jin Liu, Xia Min Wu, Dafang Chen, Xun Wang, Ning Ma, Yan Wang, Byron Good, Hong Ma, Xin Yu, Mary-Jo Good

**Affiliations:** 1 Peking University Sixth Hospital, Peking University Institute of Mental Health, Key Laboratory of Mental Health, Ministry of Health (Peking University), Beijing, China; 2 School of Public Health, Peking University, Beijing, China; 3 Department of Global Health and Social Medicine, Harvard Medical School, Boston, Massachusetts, United States of America; Institute of Psychiatry, UNITED KINGDOM

## Abstract

**Background:**

In 2005, China implemented a demonstration program known as “686” to scale-up nation-wide basic mental health services designed to improve access to evidence-based care and to promote human rights for people with severe mental disorders. As part of the 686 Program, teams “unlocked” and provided continuous mental health care to people with severe mental disorders who were found in restraints and largely untreated in their family homes. We implemented a nation-wide two-stage follow-up study to measure the effectiveness and sustainability of the “unlocking and treatment” intervention and its impact on the well-being of patients’ families.

**Methods:**

266 patients unlocked from 2005 in “686” demonstration sites across China were recruited in Stage One of the study in 2009. In 2012, 230 of the 266 cases were re-interviewed (the Stage Two study). Outcome measures included the patient medication adherence and social functioning, family burden ratings, and relocking rate. We utilized pre-post tests to analyze the changes over time following the unlocking efforts.

**Results:**

96% of patients were diagnosed with schizophrenia. Prior to unlocking, their total time locked ranged from two weeks to 28 years, with 32% having been locked multiple times. The number of persons regularly taking medicines increased from one person at the time of unlocking to 74% in 2009 and 76% in 2012. Pre-post tests showed sustained improvement in patient social functioning and significant reductions in family burden. Over 92% of patients remained free of restraints in 2012.

**Conclusion:**

Practice-based evidence from our study suggests an important model for protecting the human rights of people with mental disorders and keeping them free of restraints can be achieved by providing accessible, community based mental health services with continuity of care. China’s “686” Program can inform similar efforts in low-resource settings where community locking of patients is practiced.

## Introduction

Mental disorders constitute a huge global burden of disease, and there is a large treatment gap, particularly in low and middle-income countries (LMICs). In China, mental and behavioral disorders contribute to 23.6% of years lived with disability (YLDs) [1]. Recognizing the gap between the population’s needs for mental health care and the level of resources and services available [2–4], the Chinese government invested in an innovative reform of mental health services [5] and mental health legislation [6–7].

In 2004, as part of an effort to rebuild the public health infrastructure following the severe acute respiratory syndrome (SARS) outbreak, China officially placed mental health services within the public health system. In 2005, the government supported the launching of the National Continuing Management and Intervention Program for Psychosis, known as “686” after initial funding of CNY 6.86 million [5,8–10]. This program has contributed to improving care for patients with severe mental disorders, including schizophrenia, psychosis, bipolar disorder and schizoaffective disorder, through increasing access to treatment and integrating hospital and community services designed to provide continuity of evidence-based care and to address patients’ rights.

The 686 Program prioritized equal access to a basic package of community based mental health care for patients with severe mental illness, especially for those living in poverty. The program focused on promoting rehabilitation and recovery, family education and support, and making medications continuously available [11]. According to Liu and colleagues [5], implementing the 686 Program has led to significant progress in scaling up mental health services in China. By 2005, 60 demonstration sites had been established, two in each of China’s 30 provinces, covering 43 million people in “686” catchment areas. By the end of 2009, 96.88 million people from the general population in 112 cities were covered by this program, a total of 161,800 patients (0.17% of the general population) were registered, and 42,400 patients received regular follow-up [5].

As the 686 Program was being initiated, teams found seriously mentally ill individuals who were physically restrained or “locked” by family members accounted for approximately 0.2% of the patients registered in the “686” demonstration sites. Although these were rare events in the community, the patients indeed lived miserable lives, without care from medical personnel. Many were secluded in separate rooms or outbuildings, such as huts; some were also restrained by ropes, belts or chains; a few were locked in iron cages. Program staff used “free-medication” funds, which were developed by 686 Program for providing a subsidy (CNY 700 per case per year) for the expenses of essential antipsychotic medication to the patients who were socio-economically disadvantaged, to unlock and treat the patients in restraints between 2005 and 2008. From 2008, the 686 Program provided additional funds (CNY 5,000 per case per year) for the “unlocking and treatment” intervention to “free” the locked patients and to provide immediate medical care and enrollment in “686” follow-up services.

Widespread reports by journalists, human rights activists, and mental health specialists make it clear that in societies with limited mental health resources in Asia and Africa, many persons with severe mental illness are locked by family members and live in appalling conditions [12–16]. Others are locked or chained in temples or compounds of traditional healers [17]. Despite such reports, we find no report of empirical studies of the long-term effectiveness of programs designed to “unlock” the patients and provide mental health services in such settings. Only one report published in English demonstrated that 23 restrained patients with schizophrenia-spectrum disorder exhibited clinical improvement within 19 months of psychiatric treatment after unlocking in Bali, Indonesia [18]. However, no specific outcome measures were clearly indicated.

The present study aims to measure the long-term effects of an “unlocking and treatment” intervention undertaken from 2005 in the context of a larger mental health services reform program in China. Patients with severe mental disorders were followed-up about their medication adherence, mental health status, social functioning and family burden in 2009 and 2012 to investigate the changes over time following the unlocking efforts. We hypothesized that the patients and families would benefit from the intervention and the improvements achieved could be largely sustained through 2012. This report concludes by suggesting the implications of this intervention for other settings with limited mental health resources.

## Materials and Methods

### Participants and data collection

We designed a nation-wide two-stage follow-up study to investigate the initial phases of the 686 Program “unlocking and treatment” intervention. Individuals who fulfilled the following criteria were included in the study: 1) met diagnostic criteria for Schizophrenia, Schizoaffective Disorder, Bipolar Disorder, Delusional Disorder, Mental Retardation with psychotic symptoms, or Schizophrenia-like Psychosis in Epilepsy; 2) received the “unlocking and treatment” intervention provided by the 686 Program between January 1, 2005 and July 31, 2009; and 3) were not inpatients at the time of investigation.

**Fig 1:**
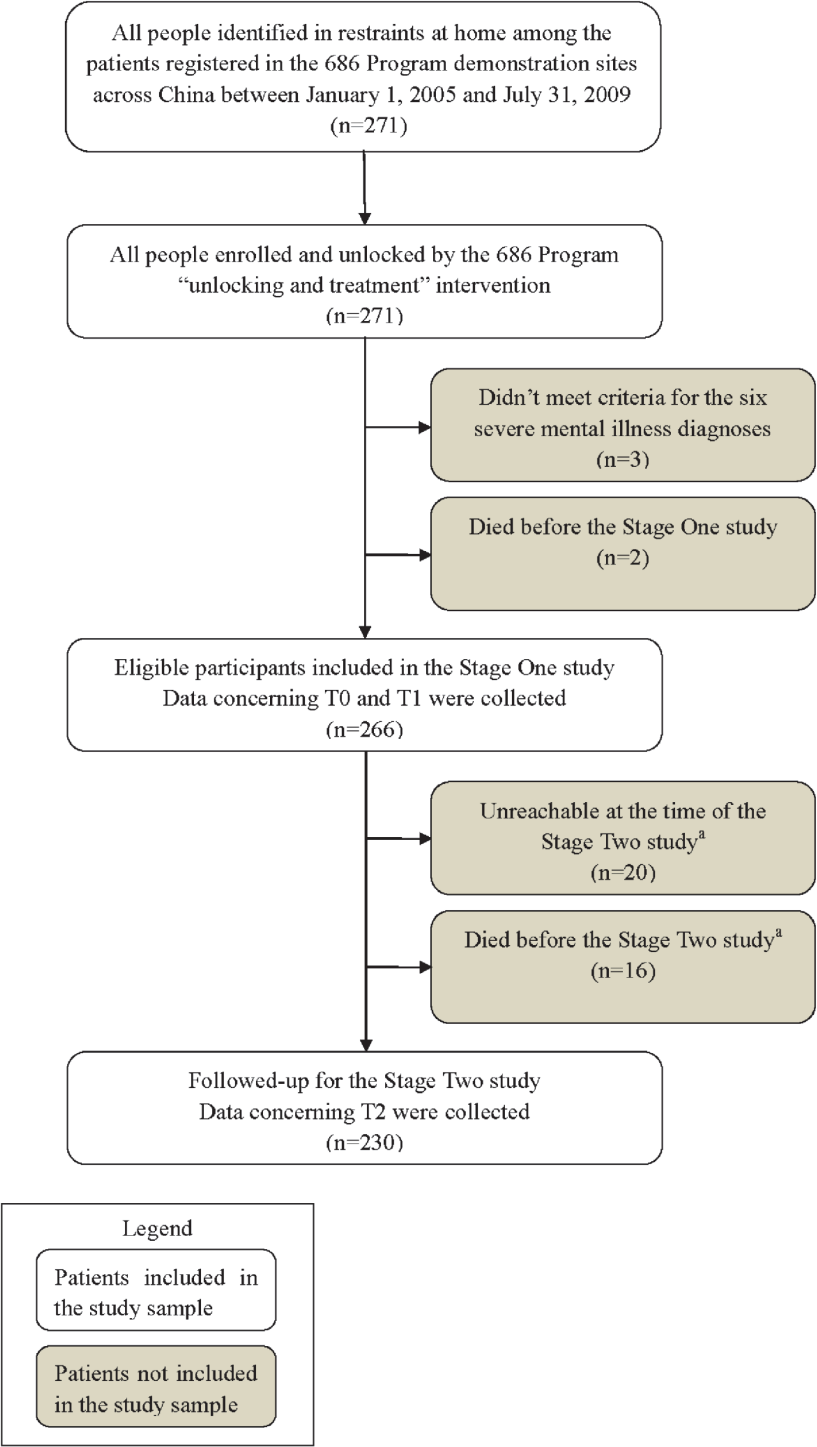
Study profile. ^a^36 patients who were not included in the Stage Two study aged from 18 to 61 (mean 40.1 [SD 12.1]). 31 (86%) were male and 28 (78%) were from rural area. 20 (56%) were unreachable due to moving and other reasons.

By July 2009, the 686 Program had enrolled and unlocked a total of 271 patients in the demonstration sites across 26 provinces of China. (No locked cases were reported by the demonstration sites in Beijing, Shanghai, Jiangsu and Zhejiang at that time.) These represented a complete sample of the locked individuals among the patients with severe mental disorders registered in “686” demonstration sites at that moment. Among them, 266 were included in Stage One of the study between August 22, 2009 and September 5, 2009. Information about patients and families were gathered for both the time when patients were unlocked (T0) and the time of Stage One interviews (T1) to allow pre-post comparison. In June 2012, 230 of these patients and families were followed-up in Stage Two of the study (T2) (Fig 1).

Case report forms designed by the “686” implementation and evaluation research team from Peking University Institute of Mental Health (PKUIMH) were utilized at both 2009 Stage One data collection (concerning T0 and T1) and 2012 Stage Two data collection (T2) to collect the same data at each time point. T0 was defined as one month prior to the “686” unlocking action; T1 was defined as the month prior to participation in the Stage One of the study in 2009; and T2 was defined as the month prior to the date of participation in the Stage Two study in 2012.

One or two key investigators from each province were trained to collect patient clinical data concerning T0, T1 and T2 from existing medical records routinely recorded by clinical staff for each unlocked patient, dated from the time patients were first unlocked by the 686 Program (i.e., T0). Hospital psychiatrists and community case managers reviewed the accuracy of the case records from hospitalizations as well as records from the community clinics. In addition, the key investigators were trained to interview family members in their homes during Stage One (concerning T0 and T1) and Stage Two (T2) studies to collect data about the locking and relocking history as well as family members’ subjective experiences of family burden.

### Ethics statement

The study was approved by the ethics review board at Peking University Institute of Mental Health (PKUIMH). Patients and/or their family members/guardians consented to participate in a written form.

### Procedure of the “unlocking and treatment” intervention

Neighborhood/village committee staff, who were trained by 686 Program to be mainly responsible for helping find the patients and leading community advocacy, provided information about the seriously mentally ill individuals who were locked in their family homes and assisted the unlocking team reach the patients. The unlocking process began with requesting permission from families of locked patients to allow a small group of trained professionals to free the patients. Among the patients registered by 686 Program, 271 were in restraints at home, and all of the families agreed to participate in the “unlocking and treatment” intervention program. The unlocking team consisted of three or more staff, including psychiatric health professionals (psychiatrists and psychiatric nurses), community persons who were often health workers, and neighborhood committee members.

Following unlocking, patients received physical exams and psychiatric diagnoses. Patients assessed to be in need of hospitalization were admitted immediately to the provincial psychiatric hospitals. Costs of medical and psychiatric treatment, including medications, were covered by the 686 Program. Upon discharge, patients were enrolled in “686” community services and followed monthly in their community by specially trained “686” case managers, who are mainly primary health care workers.

Persons unlocked who were assessed as not needing hospitalization were enrolled in “686” community services immediately after being freed; costs of antipsychotic and other prescribed medications, regular follow-ups, and community-based rehabilitation were covered by the 686 Program. As an important component of the package of care provided by the 686 Program, health education and psychosocial support were also provided to patients and their family members during follow-up visits at community health centers or village clinics. Psychiatrists, psychiatric nurses, lay health care providers and non-health workers made up multidisciplinary teams to provide mental health services and non-professional services. Changes in patients were assessed and recorded on a regular basis on follow-up by staff who were trained by the 686 Program to conduct standardized clinical assessments. These procedures continue into the present.

In case that there is failure of preventing “relocking”, the “686” teams would take immediate action to restart the “unlocking and treatment” intervention as far as possible.

### Assessments and outcome measures

#### Mental health status, diagnoses, and violent behaviors

Following unlocking, a psychiatrist conducted interviews with the patient and a family member who best knew the patient. Overall psychiatric symptoms were assessed with the Brief Psychiatric Rating Scale (BPRS) and global severity was assessed with Clinical Global Impression (CGI) scales. A final diagnosis, based on ICD-10 criteria, was made by a senior psychiatrist who reviewed the medical history, illness symptoms and associated impairments, and other available information. Violent behaviors related to initiating restraint were evaluated on level 1 to level 5 by using the scale established by the national working group of 686 Program [19], defined as follows: Level 1: threats other people verbally, or shouts at others, but doesn’t damage any physical issue; Level 2: damages only domestic property at home, and can be persuaded to stop; Level 3: damages property at home or out of home, but cannot be persuaded to stop; Level 4: repetitively damages property or hurt other people at home or out of home, but cannot be persuaded to stop; Level 5: hurts other people with any kind of weapon, or sets fire, or explodes a bomb, etc. In the 686 Program, level 3 and above is regarded as cases with high violence.

#### Medication adherence

Adherence with antipsychotic medication over the previous month was rated by the clinician in closest contact with the patient and recorded on a three point measure as good adherence, uses antipsychotics intermittently, and does not use medications. “Good adherence” was defined as patients actively take medications as prescribed, which includes following the prescribed dosing schedule, taking the full dose and refilling prescriptions. Patients were rated as “uses antipsychotics intermittently” if they followed the prescription about half of the time during last month. “Does not use medications” was defined as patients who did not receive antipsychotic medications for longer than two weeks. The rating was based on knowledge of the patient from routine clinical contact.

#### Social functioning

Assessments of social functioning include five categories: daily life activities, housework, productive labor or work, learning ability, and interpersonal relationships. Each category of social functioning was evaluated on a three point measure as good, average or poor [19]. The assessments were based on clinician’s observation of the patient’s performance as well as reports from patient and family members.

#### Family burden ratings

We explored the impact of patient mental illness on the well being of families, taking changes in caregivers’ ratings of their family burden experiences as a subjective measure. Family members were asked to rate their subjective experiences on analogue scales from 0 “no impact at all” to 10 “extremely negative impact” for the five categories of family burden—stigma, psychological pressures, economic burden, loss of personal energy, and interpersonal relationships. Ratings concerning T0 were obtained by families’ retrospective reflections at 2009 Stage One study.

#### Relocking rate

Patients remaining free of restraints after receiving “686” unlocking and treatment intervention was considered a sign of success of the program. The relocking rate was defined as the proportion of patients who were relocked after the initial “unlocking and treatment” intervention.

### Statistical analysis

The comparison of variables at T0 (before unlocking), T1 (after unlocking 2009) and T2 (after unlocking 2012) was performed using Wilcoxon signed rank tests, as the data about medication adherence and social functioning were of ordinal categorical variables, and the data about family burden ratings were not normally distributed and did not meet the criteria of compound symmetry. Chi-square tests were used to measure male/female and rural/urban differences in terms of psychiatric diagnosis and duration of illness. The level of statistical significance was defined *a priori* as a two-sided p-value of 0.05 or less. All calculations were performed with SPSS version 13.0.

As 36 (14%) cases of the total sample dropped out of the study at T2, data from 230 (86%) cases were used in the statistical analysis and related comparisons at T2. Based on our analysis of demographic features, restraint history, and measures of mental health status, violent behaviors, and social functioning at both T0 and T1, there was no evidence of systematic bias introduced by loss to follow-up of 36 patients.

## Results

### Sample characteristics

A total of 266 subjects aged 17–78 (mean age 38.6 [SD 9.9]) were included in the study at Stage One. As presented in Table 1, the “locked” population suffered from grave mental illnesses of long duration, with 96% afflicted with schizophrenia. There were no male/female and rural/urban differences in terms of psychiatric diagnosis or duration of illness (p>0.05).

**Table 1:** Demographic and disease characteristics of 266 patients at the time of inclusion for Stage One study (T1).

		Number (%)
Demographic		
**Age (years)**	≤30	53 (20%)
	>30–45	148 (56%)
	>45–60	60 (23%)
	>60	5 (2%)
**Gender**	Male	182 (68%)
	Female	84 (32%)
**Residence**	Rural	213 (80%)
	Urban	53 (20%)
**Ethnicity**	Han	222 (83%)
	Minority	44 (17%)
**Education**	Primary school and under	102 (38%)
	Junior high school	125 (47%)
	Senior high school and above	39 (15%)
**Marital status**	Single	167 (63%)
	Married	53 (20%)
	Divorced	33 (12%)
	Widowed	11 (4%)
	Remarried	2 (<1%)
**Time between T0 and T1 (years)**	≤1	96 (36%)
	>1–2	88 (33%)
	>2–3	47 (18%)
	>3–4	30 (11%)
	>4–5	5 (2%)
**Disease Characteristics**		
**ICD-10 diagnosis**	Schizophrenia	255 (96%)
	Bipolar Disorder	4 (2%)
	Mental Retardation with psychotic symptoms	4 (2%)
	Schizophrenia-like Psychosis in Epilepsy	2 (<1%)
	Schizoaffective Disorder	1 (<1%)
**Duration of illness (years)**	≤2	6 (2%)
	>2–5	23 (9%)
	>5–10	53 (20%)
	>10–20	123 (46%)
	>20–30	56 (21%)
	>30	5 (2%)

Table 2 describes the restraint history of these patients before being unlocked (T0). Total time of restraint varied from two weeks to 28 years, with 32% being locked multiple times. The vast majority of families reported that the top two reasons for “locking” a mentally ill family member were financial problems associated with caring for the patient (96%) and serious difficulties in finding capable caretakers (87%). Families also reported that patient violence triggered the most recent restraint; 94% of patients exhibited serious violent behaviors, at level 3 or above on the 686 Program’s violent behavior scale [19].

**Table 2:** Restraint history before being freed by 686 Program (T0).

		Number (%)
**Times of restraint**	1	182 (68%)
	2	39 (15%)
	≥3	45 (17%)
**Cumulative length of restraint (years)**	≤1	92 (35%)
	>1–3	82 (31%)
	>3–10	76 (29%)
	>10	16 (6%)
**Reasons for the latest restraint**	Financial difficulties	255 (96%)
	No capable care-giver	231 (87%)
	Loss of confidence in treatment	180 (68%)
	Lack of knowledge of mental illness	173 (65%)
	Fear of being known by others	41 (15%)
	Blind faith	33 (12%)
	Other reasons	12 (5%)
**Violent behavior related to initiating the latest restraint**	Level 1	3 (1%)
	Level 2	12 (5%)
	Level 3	55 (21%)
	Level 4	144 (54%)
	Level 5	52 (20%)
**Modes of the latest restraint**	Isolated in a room in the house	203 (76%)
	Iron chains used	99 (37%)
	Rope used	58 (22%)
	Isolated at a separate shed outside	35 (13%)
	Iron cage used	15 (6%)
	Other forms	2 (<1%)
**Length of the latest restraint (years)**	≤1	127 (48%)
	>1–3	58 (22%)
	>3–10	67 (25%)
	>10	14 (5%)
**Injury or poor physical health due to restraint**	Yes	57 (21%)
	No	209 (79%)

After being unlocked by the 686 Program, 233 patients (88%) were admitted to a psychiatric hospital for professional treatment; 33 patients (12%) were immediately enrolled in “686” community outpatient programs. Upon discharge from psychiatric hospital, 91% of inpatients were regarded by medical staff as improved and 9% as significantly improved; two (<1%) showed no improvement.

### Changes in patient medication adherence and social functioning

Table 3 illustrates changes in patients as measured in Stage One (T0 and T1) and Stage Two (T2) studies. Prior to unlocking, 82% of the patients were not receiving antipsychotic medication; 17% of the patients were receiving medications and used them intermittently; only one patient showed good adherence to medication. All 266 patients were assessed to require antipsychotic medications at the time of unlocking.

**Table 3:** Changes in patient medication adherence and social functioning (T0, T1 and T2 comparison).

	T0	T1	T2	T0-T1 test	T0-T2 test	T1-T2 test
	(n = 266)	(n = 266)	(n = 230)	(n = 266)	(n = 230)	(n = 230)
				p-value	p-value	p-value
**Medication Adherence**						
Good adherence	1 (<1%)	197 (74%)	175 (76%)	<0.0001	<0.0001	0.2032
Uses antipsychotics intermittently	46 (17%)	59 (22%)	33 (14%)			
Does not use medications	219 (82%)	10 (4%)	22 (10%)			
**Social Functioning**						
**Daily life activities**						
Good	0 (0%)	64 (24%)	55 (24%)	<0.0001	<0.0001	0.0065
Average	8 (3%)	182 (68%)	133 (58%)			
Poor	258 (97%)	20 (8%)	42 (18%)			
**Housework**						
Good	0 (0%)	29 (11%)	33 (14%)	<0.0001	<0.0001	0.0123
Average	6 (2%)	187 (70%)	124 (54%)			
Poor	260 (98%)	50 (19%)	73 (32%)			
**Productive labor or work**						
Good	0 (0%)	15 (6%)	18 (8%)	<0.0001	<0.0001	0.2452
Average	1 (<1%)	136 (51%)	100 (43%)			
Poor	265 (>99%)	115 (43%)	112 (49%)			
**Learning ability**						
Good	0 (0%)	4 (2%)	6 (3%)	<0.0001	<0.0001	0.003
Average	0 (0%)	138 (52%)	90 (39%)			
Poor	266 (100%)	124 (47%)	134 (58%)			
**Interpersonal relationships**						
Good	0 (0%)	14 (5%)	8 (3%)	<0.0001	<0.0001	<0.0001
Average	4 (2%)	151 (57%)	101 (44%)			
Poor	262 (98%)	101 (38%)	121 (53%)			

Data are number and % unless otherwise stated.

Significant changes in medication adherence occurred after patients were unlocked and entered into the “686” treatment program. Three quarters of patients were assessed as having good adherence to medication treatment both at T1 and at T2. Social functioning in each of the five categories also improved significantly, showing major changes from T0 to T1 (p<0.0001). Although the proportion of patients with poor functioning increased slightly from T1 to T2 (p<0.05) except in “productive labor or work” category (p = 0.2452), most changes were sustained through 2012, as indicated by the T0-T2 comparisons (p<0.0001).

### Changes in family burden ratings

As illustrated in Table 4, when comparing family members’ evaluations on how much they were affected by the patients’ mental illness, 2009 post-unlocking scores illustrated significant improvement in caregivers’ experience with a reduction in rating scores on each of the measures of family burden from T0 to T1 (p<0.0001). Although measures of family burden except stigma (p = 0.1089) increased slightly from T1 to T2 (p<0.0001), the improvement in five categories of family burden ratings were largely sustained as shown in the T0-T2 comparisons (p<0.0001).

**Table 4:** Changes in family burden ratings (T0, T1 and T2 comparison).

	T0^a^	T1	T2	T0-T1 test	T0-T2 test	T1-T2 test
	(n = 266)	(n = 266)	(n = 230)	(n = 266)	(n = 230)	(n = 230)
				p-value	p-value	p-value
**Stigma**	8.4 (2.0)	4.3 (1.7)	4.6 (2.3)	<0.0001	<0.0001	0.1042
**Psychological pressures**	8.9 (1.4)	4.5 (1.7)	5.3 (2.3)	<0.0001	<0.0001	<0.0001
**Economic burden**	9.2 (1.2)	4.9 (2.1)	6.2 (2.6)	<0.0001	<0.0001	<0.0001
**Personal energy**	8.9 (1.4)	4.6 (1.8)	5.5 (2.2)	<0.0001	<0.0001	<0.0001
**Interpersonal relationships**	8.7 (1.7)	4.6 (2.0)	5.4 (2.4)	<0.0001	<0.0001	<0.0001

Data are mean (SD). Family members were asked to rate their subjective experiences on analogue scales from 0 “no impact at all” to 10 “extremely negative impact”. The higher the score the higher the family members experience family burden that was associated with patients’ mental illness.

^a^Ratings concerning T0 were obtained by families’ retrospective reflections at 2009 Stage One study.

### Relocking rate

By the time of the Stage Two study, 21 (8%) of the initial 266 individuals had an episode of “relocking” following the “686” intervening efforts. Poor adherence to treatment, including “uses antipsychotics intermittently” and “does not use medications”, was found at T2 in 12 (57%) cases who were relocked. Families noted the top two reasons for relocking to be similar to reasons for initial locking: no capable care-giver was available and financial difficulties.

## Discussion

This study reports findings of an implementation research project designed to measure the effectiveness of an “unlocking and treatment” intervention that was developed in the context of China’s 686 Program [10]. Our analysis of cases of patients who had been unlocked, entered into treatment, and returned to their homes—266 at baseline in 2009, and 230 in 2012—provides the first practice-based evidence for long-term benefits of an intervention approach that is linked to a major mental health services reform.

### Positive and sustainable impact of the intervention

The persons locked by family members in rural and urban China were primarily patients who had lived with severe mental illness for many years, as a result of a functional failure of the traditional hospital-based mental health model. Less than 1% of the total sample were in regular treatment. Unlocking patients and providing access to free hospital treatment and continuing mental health care in the community were shown to have powerful and sustained benefits for these patients. It is encouraging that the number of persons regularly taking medicines increased from one person at the time of unlocking to three quarters in 2009 and in 2012, especially given that an epidemiological study suggests only 60% of persons with psychotic illness in China have ever sought specialized mental health care [4].

Besides the improved clinical outcome achieved, improved social functioning was shown to hold not only through 2009 (T1) but through 2012 (T2), compared with the time the patients were freed by the 686 Program (T0). Improved access to care and social functioning of patients had direct benefits for the lives of families as well. Family caregivers described significant reductions in feelings of stigma, psychological pressures, economic burden, loss of personal energy, and negative personal relationships at both T1 and T2.

We observed some decline in patient social functioning and increased family burden from T1 to T2. This might be due to the habituation effect. Over the years after the “unlocking”, families became accustomed to the initial impact of the initiative on patients’ clinical condition, and thereby their ratings were biased toward minimizing the initial improvement at T2. Other possible explanations might be: 1) Patients received more intensive services during T0-T1, especially immediately after being enrolled in the “unlocking and treatment” intervention, than during T1-T2; such services included inpatient care, assistance from the Disabled Federation and the Civil Affairs Association, the “686” rehabilitation programs, etc. 2) Those were the most vulnerable population in the community with the least societal resources. Therefore the families had very limited ability to take care of the patients and cope with family burdens. It is suggested that further efforts are needed to continue to support the long-term recovery of patients from severe mental illness and to improve the well being of the families.

### Rights promotion: unlocking patients through scaling up mental health care

Our study suggests that successful eradication of restraint practices can be achieved and sustained by scaling up services to provide effective, accessible and affordable care for people living with mental disorders [20–21]. The finding that more than 92% of those unlocked and entered into continuous treatment by the 686 Program remained free of restraints by 2012 demonstrates the feasibility of improving the human rights of persons with severe mental illness by increasing access to mental health care in the community [22], even with limited societal resources. Nevertheless, the failure to prevent relocking for 21 individuals suggests that considerable room for improvement of our mental health care practice still exists. Continuous and efficient treatment and strong social supports are warranted to prevent the recurrence of restraints.

Persons with severe mental illness worldwide live in “zones of abandonment” [23]. In North America, these include the streets, halfway houses, homeless shelters, and prisons. In countries with very limited mental health resources, these are often homes of family members, where a combination of lack of medical care, outbursts of violence inside the household or a tendency to wander outside the house, stigma and embarrassment, and lack of ability of families to provide care lead to seclusion and restraints [24]. This phenomenon, which occurs throughout the world [15–18,25–27], represents human rights violations of individuals with mental and psychosocial disabilities [20]. To prevent patients with severe mental disorders from being pushed to the margins of society, continuous development of accessible services in the community should be guaranteed for the long run. Policies and laws should be well formulated to stimulate advocacy and education campaigns, as well as establish and develop legal and oversight mechanisms to prevent human rights violations [7,20].

### Limitations

Limitations of this study grow out of the fact that it was designed after the program was underway; it is not a prospective study with an experimental design. Family burden ratings at T0 were obtained by family care-givers’ retrospective reflections, which may introduce bias; these findings should therefore be interpreted with caution given their subjective nature. However, family care-givers, usually parents and spouse of the patients, had very clear memories concerning the unique and painful experience of locking a mentally ill family member. We believe that with limitations, the rating of family burden based on retrospective recall thus has important validity. The sample of the study was 96% schizophrenia; this might make it difficult to generalize findings to locked individuals with other serious mental illnesses.

At the time of this study, it was possible that one or more patients with severe mental disorders remained locked and were not reached by the 686 Program during implementation in the demonstration sites. The 686 Program was implemented according to the annual budget and tasks allocated by the central government, and patients who were the most mentally ill and lived in poverty were prioritized. The patients registered at the initial phase of the 686 Program were 0.17% of the general population, and 0.2% of all those registered were found to be in restraints and therefore freed and treated by the Program. In a few cases, although the exact number was not available, patients or families refused to be enrolled by 686 Program for some concerns. Further efforts are warranted for the Program to cover the whole population of patients with severe mental disorders and provide effective mental health care for people in need. With such efforts, it is hoped that those individuals who remain in restraints at home can be fully identified, and “locking” of patients can be ultimately eliminated.

## Conclusions

There is obviously much to be done in China to scale up the 686 Program for the whole nation [28–29] and to improve quality of care. This will require substantially increased investment in mental health services. However, the success of the program in maintaining the severely ill individuals in treatment over three to seven years, and the benefits of the intervention for those who have lived for years with untreated psychosis and for their families, attests to the feasibility and social value of the “686” model.

The Chinese “686” model can serve to inform similar efforts in other LMICs where restraint of the patients continues [15–18,25–27]. Extending protection of human rights of persons with severe mental illness and providing patients equitable access to effective care in the community remain enormous challenges in the least resourced countries [30–31]. Findings of this study suggest that the Chinese model may be one of the important models for low-resource settings. The 686 program demonstrates the feasibility of building model mental health programs to scale up services [21,29], by using limited investment of central government to mobilize administrative and societal resources, allocating funds from local government and lay organizations, as well as by involving lay health care providers and non-health workers in the multidisciplinary mental health service team [5,32].
